# Diffuse Neuroendocrine Cell Hyperplasia: Report of Two Cases

**DOI:** 10.1155/2016/3419725

**Published:** 2016-05-17

**Authors:** Cevriye Cansız Ersöz, Ayten Kayı Cangır, Serpil Dizbay Sak

**Affiliations:** ^1^Department of Pathology, Faculty of Medicine, Ankara University, Sıhhiye, 06100 Ankara, Turkey; ^2^Department of Thoracic Surgery, Faculty of Medicine, Ankara University, Ankara, Turkey

## Abstract

Diffuse idiopathic pulmonary neuroendocrine cell hyperplasia (DIPNECH) is a rare pulmonary disorder characterised by a proliferation of neuroendocrine cells within the lung. It is believed that a minority of the patients with DIPNECH can develop carcinoid tumors. Here, we report two new cases of DIPNECH with coexisting carcinoid tumors.

## 1. Introduction

Neuroendocrine (NE) cells are a component of pulmonary epithelium. NE cell hyperplasia may be reactive or idiopathic. Reactive NE cell hyperplasia may be seen in various conditions characterised by hypoxia. Idiopathic type is defined as diffuse idiopathic pulmonary neuroendocrine cell hyperplasia (DIPNECH). A minority of DIPNECH patients can develop carcinoid tumors. Herein, we would like to document two additional cases of carcinoid tumors in the background of DIPNECH.

## 2. Cases

### 2.1. Case 1

**Figure 1:**
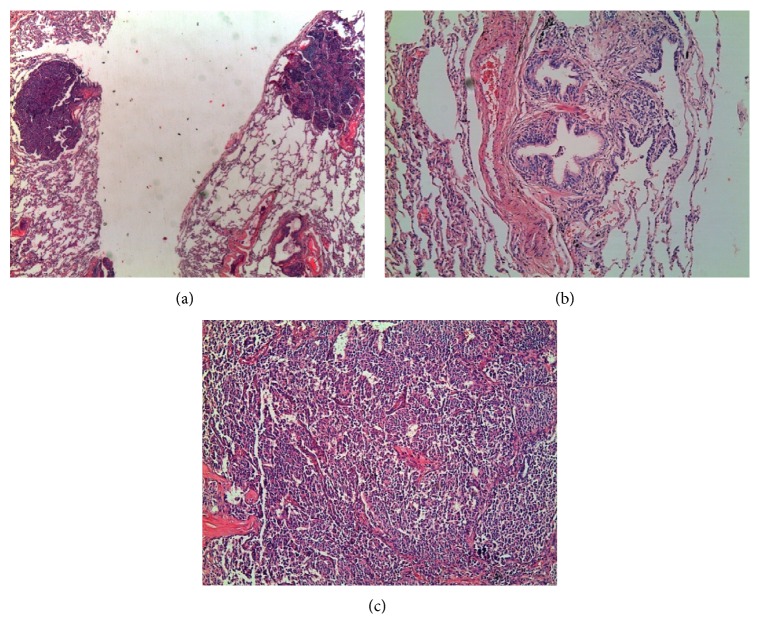
(a) Tumorlets HE ×2.5, (b) NE cell hyperplasia HE ×10, and (c) carcinoid tumor HE ×10.

51-year-old, nonsmoker female was admitted to hospital because of cough and hemoptysis. She had a history of several hospital admissions during the previous decade, due to lung infection, and she was being treated for asthma since 4 years. A hypodense nodule, measuring 1.7 cm, was identified in hilar region on thoracic CT and a wedge resection of left lower lobe was performed. Histopathological examination showed two foci of typical carcinoid tumors, multiple tumorlets and widespread neuroendocrine hyperplasia. Carcinoid tumors and tumorlets were characterised by monotonous tumor cells with round/ovoid nuclei with stippled chromatin, indistinct nucleoli, forming nests, and trabeculae (Figure 1). Lung parenchyma in between the neuroendocrine cells and tumors was entirely normal. On immunohistochemical examination, all lesions were positive with Chromogranin A (ChrA), Synaptophysin (SYNP), and Neuron Specific Enolase (NSE). After 7 years of uneventful follow-up, multiple lung nodules, the largest measuring 1.1 cm, probably representing new carcinoid tumors, were identified, on thoracic CT. However, the patient was lost to follow-up at this stage and histological examination could not be performed.

### 2.2. Case 2

**Figure 2:**
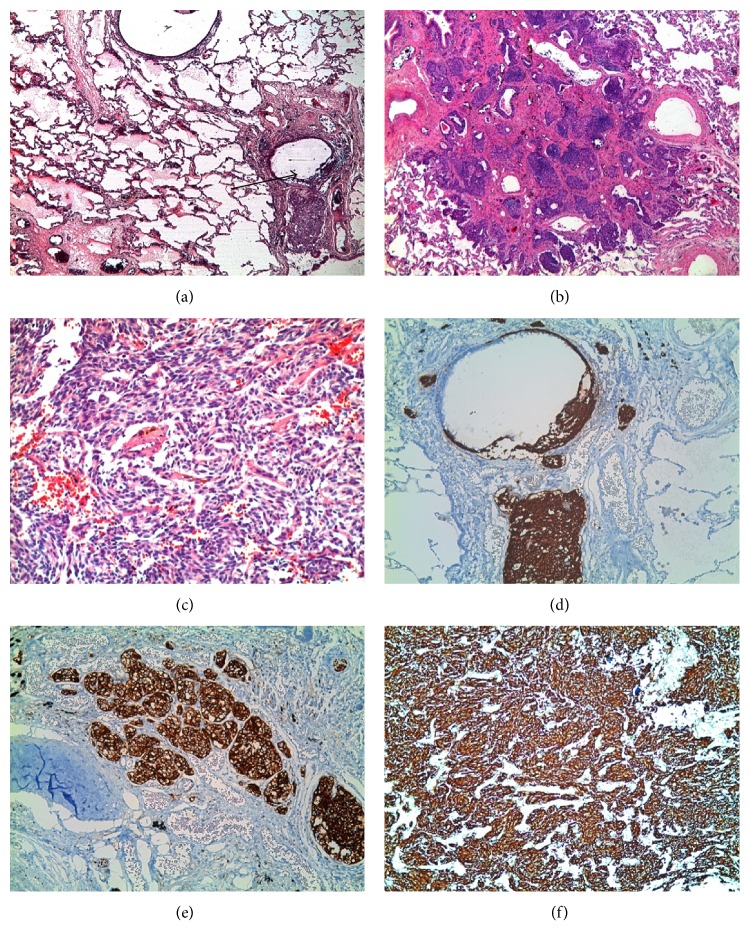
(a) Linear and nodular proliferation of neuroendocrine cells within terminal bronchiole submucosal layer and tumorlets (HE ×2.5). (b) Tumorlet (HE ×4). (c) Carcinoid tumor (HE ×20). (d) ChrA reactivity of the same area in (a) (ChrA ×10). (e) CD56 positivity in a tumorlet (CD56 ×10). (f) Carcinoid tumor cells with strong SYNP positivity (SYNP ×10).

66-year-old, nonsmoker female, scheduled for cataract surgery, had a pulmonary nodule on routine chest X-ray examination. On thoracic CT, a nodule measuring 1.5 cm was identified in the right middle lobe and lobectomy was performed. Tumor was composed of spindle cells with round to ovoid nuclei, coarse chromatin, indistinct nucleoli, and eosinophilic cytoplasm within a hyalinized stroma. The tumor was diagnosed as a typical carcinoid tumor. There were multiple tumorlets and neuroendocrine cell hyperplasia in the background. All lesions were positive for ChrA, SYNP, and CD56 (Figure 2).

**Figure 3:**
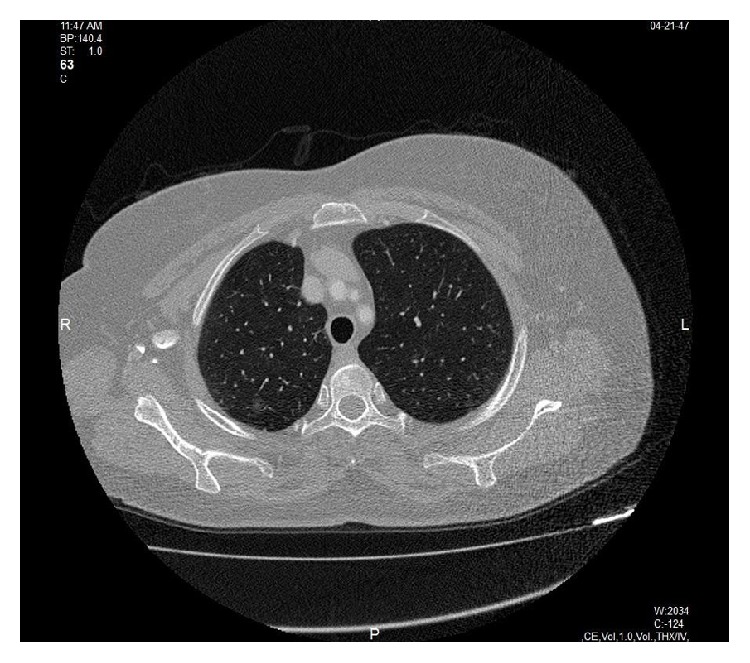
CT examination of the second patient, after 2 years from the lung operation.

There were no other pathologic findings in the lung parenchyma. After 2 years from the operation, she developed multiple lung nodules, the largest measuring 1 cm, on thoracic CT (Figure 3). She is still alive.

## 3. Discussion

NE cells are a component of pulmonary epithelium that comprises about 1% of all epithelial cells in an adult lung [1]. NE cell hyperplasia can be described as clusters of three or more NE cells [1]. Neuroendocrine cell hyperplasia can occur in three different settings. The first condition is a nonspecific secondary reaction to airway/interstitial inflammation and/or fibrosis that cause hypoxia. Pulmonary neuroendocrine cell (PNEC) hyperplasia can be observed in association with a wide spectrum of pulmonary conditions, particularly bronchiectasis, chronic obstructive pulmonary disease, and pulmonary interstitial fibrosis. It can be observed in cigarette smokers and in individuals living at high altitudes. This is considered as a reactive process associated with chronic damage [2, 3]. The second condition is PNEC hyperplasia observed in the mucosa of bronchi or bronchioles adjacent to carcinoid tumors. Miller and Müller found neuroendocrine cell hyperplasia in adjacent mucosa, in 76% of the carcinoid tumors [4]. In this study, hyperplasia was defined in the “adjacent” mucosa and there was no comment about the rest of the lung parenchyma. In a recent study, comparing the frequency of PNEC hyperplasia in pulmonary neuroendocrine tumours and non-neuroendocrine cell carcinomas, it was noted that there are increased levels in NE tumours (other than small cell carcinomas), compared with a control group of non-neuroendocrine non-small cell carcinomas [5].

The third condition is called diffuse idiopathic pulmonary neuroendocrine cell hyperplasia (DIPNECH) in which the hyperplasia is diffuse and primary in nature. In a recent study, “the presence of 5 or more NE cells, singly or in clusters located within the basement membrane of the bronchiolar epithelium of at least 3 bronchioles, combined with 3 or more carcinoid tumorlets” was recommended as diagnostic criteria for DIPNECH in surgical pathology specimens [6].

In contrast to reactive NE cell hyperplasia, DIPNECH is a primary disorder, often incidentally detected without preexisting chronic lung disease. Lung parenchyma adjacent to the lesions must be essentially normal, with no evidence of inflammation, fibrosis, or other chronic pulmonary diseases. But DIPNECH may be accompanied by mild, chronic lymphocytic inflammation and fibrosis of involved airways. In 1992, Aguayo et al. [3] described six patients with NE cell hyperplasia, without any predisposing conditions, and named the entity as DIPNECH. Based on this series and other descriptions, WHO included DIPNECH in the tumor classification as a preinvasive lesion [7].

The gender distribution of patients with lung carcinoid is equal, but DIPNECH is more frequent in women. According to the review by Nassar et al., 23 of the 25 cases (92%) are women, as in our two cases [8]. The mean age of diagnosis is about 60 years and like our patients, DIPNECH is more frequent in patients without a history of smoking [3, 7].

In the series by Davies et al., 10 of the 19 patients were asymptomatic [9]. Most symptomatic patients with DIPNECH present with nonproductive cough and dyspnoea. Symptoms and lung function tests may be misinterpreted as asthma. The clinical picture is thought to be correlated with peptide secretory substances. The hyperplastic neuroendocrine cells contain a variety of neuropeptides, including bombesin-like peptides; secretagogue and bronchoconstrictive effects of these peptides may play a role in the pathogenesis [10]. Fibrous obliterative bronchiolitis and peribronchiolar fibrosis are important histological features associated with proliferation of PNECs and are seen in only one-third of the cases [3, 11]. Fibrosis starts focally in the submucosa and progresses forming plaques that slowly obliterate the bronchial lumen [10]. In symptomatic patients, evidence of small airway obstruction in the form of mosaicism and the coexistence of multiple pulmonary nodules may be seen in HRCT. However absence of HRCT findings does not negate the diagnosis. In asymptomatic patients, the disease is diagnosed incidentally by thoracic imaging, performed for an unrelated condition. Lung nodules are the most frequent radiological manifestations. Multiple lung nodules generally raise the suspicion of metastases from an unknown primary site. Our first patient was symptomatic and was treated for presumed asthma. The symptoms can be attributed to the neuropeptides released from neuroendocrine cells, as there was no evidence of fibrous obliterative bronchiolitis or peribronchiolar fibrosis in this patient. Multiple lung nodules were identified in this patient, after seven years from the initial operation. It can be speculated that these nodules may be foci of tumorlets/carcinoid tumors. In our second case, lung nodule was incidentally identified on CT examination.

Histologically, DIPNECH is defined as NE cell hyperplasia confined to the respiratory epithelium without penetration through basement membrane. It can be seen as diffuse proliferation of scattered neuroendocrine cells, small nodules, or a linear proliferation of neuroendocrine cells. Extension beyond the basement membrane is called “tumorlet” if the collection of cells is smaller than 5 mm and “carcinoid” when 5 mm or larger. Although the exact frequency of carcinoid tumor development on DIPNECH is unknown, Davies et al. found 47% and Nassar et al. found 40% in their case series [8, 9]. Koo at al. suggested that these three entities represent different stages along a spectrum of neuroendocrine cell proliferation [12]. Although this model is useful, it is important to note that carcinoids can be seen without any precursor lesion.

DIPNECH usually runs an indolent or stable course but progressive disease with obliterative bronchiolitis requiring lung transplantation has been reported [9]. Our first patient was alive after 7 years from operation, with multiple small nodules in the lung on CT, but subsequent clinical status is unknown. The second patient is in the postoperative third year and in stable clinical status, with multiple lung nodules detected on CT examination.

## 4. Conclusions

DIPNECH is a rare entity. Although most of the cases described are symptomatic patients with lung function alterations, it should also be included in the differential diagnosis of asymptomatic patients with normal lung function and compatible radiological alterations, especially in middle-aged women without history of smoking.
